# FLAME, a novel fuzzy clustering method for the analysis of DNA microarray data

**DOI:** 10.1186/1471-2105-8-3

**Published:** 2007-01-04

**Authors:** Limin Fu, Enzo Medico

**Affiliations:** 1 Laboratory of Functional Genomics, The Oncogenomics Center, Institute for Cancer Research and Treatment (IRCC), University of Torino, School of Medicine, 10060 Candiolo, Italy.

## Abstract

**Background:**

Data clustering analysis has been extensively applied to extract information from gene expression profiles obtained with DNA microarrays. To this aim, existing clustering approaches, mainly developed in computer science, have been adapted to microarray data analysis. However, previous studies revealed that microarray datasets have very diverse structures, some of which may not be correctly captured by current clustering methods. We therefore approached the problem from a new starting point, and developed a clustering algorithm designed to capture dataset-specific structures at the beginning of the process.

**Results:**

The clustering algorithm is named Fuzzy clustering by Local Approximation of MEmbership (FLAME). Distinctive elements of FLAME are: (i) definition of the neighborhood of each object (gene or sample) and identification of objects with "archetypal" features named Cluster Supporting Objects, around which to construct the clusters; (ii) assignment to each object of a fuzzy membership vector approximated from the memberships of its neighboring objects, by an iterative converging process in which membership spreads from the Cluster Supporting Objects through their neighbors. Comparative analysis with K-means, hierarchical, fuzzy C-means and fuzzy self-organizing maps (SOM) showed that data partitions generated by FLAME are not superimposable to those of other methods and, although different types of datasets are better partitioned by different algorithms, FLAME displays the best overall performance. FLAME is implemented, together with all the above-mentioned algorithms, in a C++ software with graphical interface for Linux and Windows, capable of handling very large datasets, named Gene Expression Data Analysis Studio (GEDAS), freely available under GNU General Public License.

**Conclusion:**

The FLAME algorithm has intrinsic advantages, such as the ability to capture non-linear relationships and non-globular clusters, the automated definition of the number of clusters, and the identification of cluster outliers, i.e. genes that are not assigned to any cluster. As a result, clusters are more internally homogeneous and more diverse from each other, and provide better partitioning of biological functions. The clustering algorithm can be easily extended to applications different from gene expression analysis.

## Background

Data clustering is based on the assumption that a population of objects can be subdivided into smaller subgroups, internally homogeneous for one or more features. Since the work of Eisen and colleagues [1], clustering methods have become a key step in microarray data analysis, to identify groups of genes or samples displaying a similar expression profile. Such partitioning has the main scope of facilitating data visualization and interpretation, and can be exploited to gain insight into the transcriptional regulation networks underlying a biological process of interest. As an example, promoters of genes belonging to the same transcriptional clusters in a yeast cell cycle experiment were found to be enriched for specific sequence motifs, likely to serve as transcription factor binding sites [2,3]. However, due to the complex nature of biological systems, microarray datasets tend to have very diverse structures, some of them even do not seem to have well-defined clustering structures. As a result, none of the existing clustering algorithms performs significantly better than the others when tested across multiple datasets [4-6]. Commonly used algorithms, such as k-means, hierarchical clustering and Self-Organizing Maps (SOM) [7], typically construct clusters on the basis of pairwise distance between genes. As a consequence, they may fail to reveal nonlinear relationships between gene expression profiles, and thereby fail to correctly represent a dataset with nonlinear structure [8]. As a typical example of non-linear relationship, a modest shift in the time-course response of two genes is sufficient to substantially reduce any linear similarity measurement value. Moreover, K-means and SOM fail to capture non-globular clusters and to avoid solution trapping in local minima [4,9]. Over the past years, more sophisticated clustering approaches have been developed specifically for microarray data clustering, such as GeneClust [5], biclustering [10] and CLIFF [11]. Though in some particular cases they perform better than standard methods, none of them proved consistently better across multiple different datasets (reviewed in [4]). Moreover, their high algorithmic complexity severely limited their use and the traditional algorithms remain more popular thanks to their conceptual simplicity. In particular, hierarchical clustering remains the most widely used clustering algorithm, although it has been described to suffer from a number of limitations mostly deriving from the local decision making scheme that joins the two closest genes or clusters without considering the data as a whole [12].

Recently, fuzzy clustering approaches have been taken in consideration because of their capability to assign one gene to more than one cluster (fuzzy assignment), which may allow capturing genes involved in multiple transcriptional programs and biological processes. Fuzzy C-means (FCM), also named Fuzzy K-means, is a fuzzy extension of K-means clustering and bases its fuzzy assignment essentially on the relative distance between one object and all cluster centroids [13,14]. Many variants of FCM have been proposed in the past years, including a heuristic variant that incorporates principle component analysis (PCA) and hierarchical clustering [15], and Fuzzy J-Means, that applies variable neighborhood searching to avoid cluster solution being trapped in local minima [16]. A FuzzySOM approach was also developed, to improve FCM by arraying the cluster centroids into a regular grid [17]. All these fuzzy C-means-derived clustering approaches suffer from the same basic limitation of K-means, i.e. using pairwise similarity between objects and cluster centroids for membership assignment, thereby lacking the ability to capture non-linear relationships [8]. Another family of fuzzy clustering approaches is based on Gaussian Mixture Model (GMM) [18-20], where the dataset is assumed to be generated by a mixture of Gaussian distributions with certain probability, and an objective function is calculated based on the mixture Gaussians as the likelihood of the dataset being generated by such model. Then the objective function is maximized to solve the model and give a set of probabilistic assignment. A possible problem with this approach, as highlighted by Yeung and colleagues, is that real expression data not always satisfy the basic Gaussian Mixture assumption even after various transformations aimed at improving the normality of the data distributions [20].

The aim of this paper is to propose a conceptually novel clustering algorithm combining simplicity with good performance and robustness. The algorithm approaches fuzzy data clustering from a novel perspective. It is mainly based on two general assumptions: (a) clusters should be identified in the relatively dense part of the dataset; (b) neighboring objects with similar features (expression profiles) must have similar cluster memberships so that the membership of one object is constrained by the memberships of its neighbors. Therefore, the membership of each single object (gene or sample) is not determined with respect to all other objects in the dataset or to some cluster centroids, but is determined with respect to its neighboring objects only. This approach brings the notable advantage of capturing non-linear relationships, in a way similar to a nonlinear data dimensionality reduction approach called Locally Linear Embedding (LLE), originally developed for mapping multi-dimensional objects (data points) into a lower-dimension space for their representation [21]. The idea behind LLE is that, in a dataset, most nonlinear relationships can be effectively captured by subdividing the general network of relationships across all objects into locally linear relationships between neighbor objects. As an important consequence, information about one object can be correctly approximated by information obtained from its nearest neighbors. So for each object, LLE used the original dataset to define its nearest neighbors and to assign a set of weights specifying how much each neighbor contributes to the reconstruction of the features (coordinates) of the object. After this, the dataset can be represented in a lower dimensional space, where each object is mapped according to the lower dimensional representation of its nearest neighbors and the weights assigned to its nearest neighbors. In this way the local structure of the original dataset (the neighbors of each object and their proximity) is preserved also in a lower dimensional space such as the 2d or 3d views commonly used for data displaying. We therefore envisaged a fuzzy clustering approach based on neighborhood approximation, to capture non-linear relationships in multidimensional data and to provide a substantial improvement in the visualization and analysis of microarray data. The novel clustering method, FLAME, integrates the two above-mentioned key properties: (a) fuzzy membership assignment (one-to-many gene-to-cluster relationship); (b) definition of membership assignment by local approximation, where membership assignment of a gene depends on membership assignments of its neighbors genes (genes showing similar behavior).

## Results

### The FLAME algorithm

**Figure 1:**
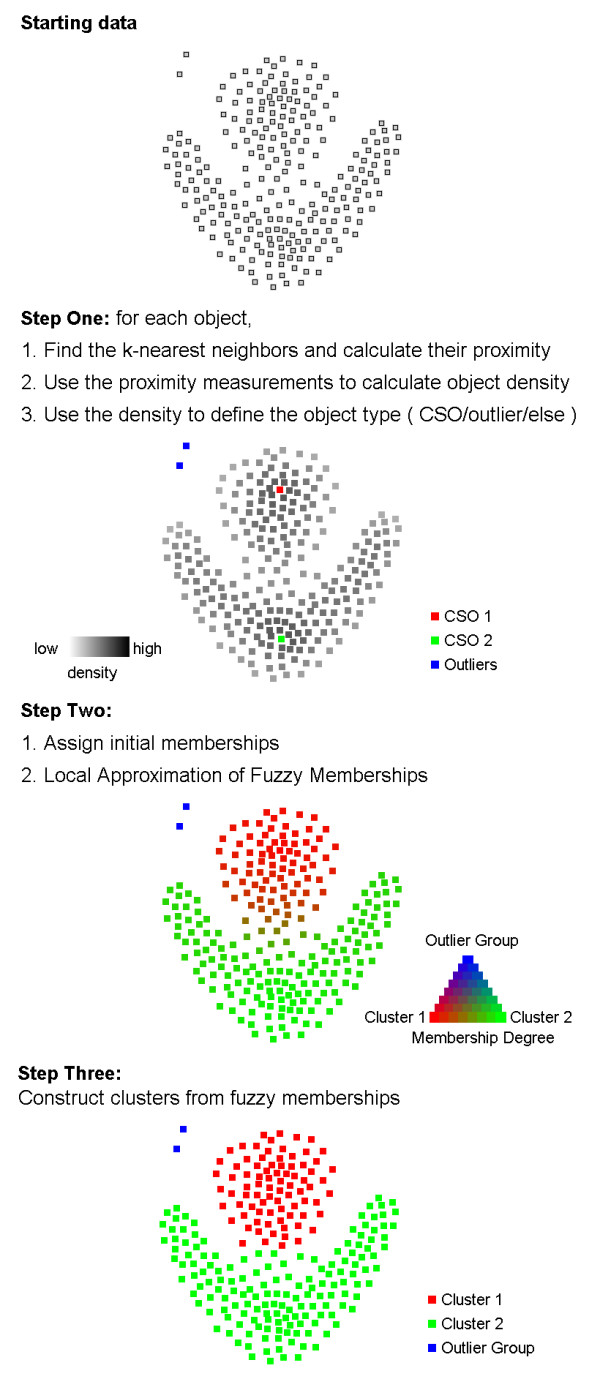
The key steps of the FLAME algorithm shown on a small simulated dataset ("Starting data"). **Step One: **expression data are used to calculate for each gene a density value corresponding to the average similarity to its nearest neighbors (in the picture, darkness of each spot is proportional to density); Cluster Supporting Objects (CSOs) are then identified as genes with local maximum density and assigned unique membership to themselves. The red and green colors define two CSOs, while the blue color indicates outliers. **Step Two: **for all the other genes, a fuzzy membership vector is approximated from the memberships of their nearest neighbors, until convergence; for each spot, red, green and blue colors are now mixed in accordance with the fuzzy membership of that gene to the two clusters or to the outlier group. **Step Three: **at the end of this process, genes can be assigned to one of the two clusters built around the CSOs or to the outlier group, based on their approximated memberships.

Data clustering by FLAME goes through three main steps, illustrated in Figure 1. The first is the extraction of local structure information and identification of cluster supporting objects (CSO's). In this step, the distance/proximity between each object and its k-nearest neighbors is used to calculate object density. Objects with the highest density among their neighbors are identified as CSOs and serve as prototypes for the clusters, based on the fact that many other objects show similar behavior. Some outliers are also identified in this step, whose behavior is rare in the dataset. The second step is the assignment of fuzzy membership by local approximation. The initial number of clusters is defined by the number of CSOs. At the beginning, each object is assigned with equal membership to all clusters, with the exception of CSOs and outlier objects, each CSO being assigned with full membership to itself as a cluster, and all outlier objects being assigned with a full membership to the outlier group. Then, an iterative process is performed to approximate the fuzzy memberships of objects which are not CSOs or outliers, for which the membership is fixed. At each iteration, the fuzzy membership of each object is updated by a linear combination of the memberships of its nearest neighbors, weighted by their proximity. In this process the fixed, full memberships of CSOs and outliers exert an influence on the membership of their neighbors, which subsequently propagates in the neighborhood network during the following iterations so that the final membership of each object (except CSOs and initial outliers) is the result of a balanced influence (direct and indirect) of the memberships of all other objects. To facilitate comprehension of the process of membership "propagation", a Flash animation is available as additional movie [see Additional file 1]. The last step is the construction of clusters from the fuzzy memberships, which can be made in two ways: (i) by assigning each object to the cluster in which it has the highest membership degree (one to one object-cluster relationship), or (ii) by applying a threshold on the memberships, and assign each object to the one or more clusters in which it has a membership degree higher than the threshold (one-to-many object-cluster relationship). In the validation analysis presented here, we used the single membership approach.

Before assessing the clustering performances of FLAME, we preliminarily estimated its computational efficiency by analyzing its time complexity [see Additional file 2]. As a theoretic time complexity estimation of the membership approximation procedure is very difficult, we performed an empirical study of the time complexity of FLAME compared with other algorithms [see Additional file 3]. The empirical result shows that for data matrices with many columns, FLAME has significant computational advantage over the other methods, except K-means. But actually in our implementation, no sophisticated techniques have been implemented for K-means to search for global minimum, while FLAME always guarantees global minimum. Taking this into account, K-means may not have much computational advantage over FLAME.

### FLAME implementation in the GEDAS software

The whole FLAME algorithm has been implemented as a part of Gene Expression Data Analysis Studio (GEDAS), a C++ program with graphical user interface currently running on Linux and Microsoft Windows. Two user modes are provided, Simple Mode, which is enough for most usages, and Advanced Mode, which enables tuning of all parameters to optimize the clustering. The key parameter to tune during FLAME optimization is the KNN number, because it affects the number of clusters in different ways. First, KNN determines the smoothness of density estimation (the number of peaks in the density distribution), which in turn limits the maximum number of CSOs. Second, KNN determines the range covered by one CSO: the larger the KNN, the larger the CSO range, with fewer CSOs. In the end, in the neighborhood approximation step, KNN determines the range of membership influence of each object: the larger the KNN, the fuzzier the memberships of the genes. Four other clustering algorithms, K-means, hierarchical clustering, Fuzzy C-Means (FCM) and Fuzzy SOM (FSOM) are implemented in GEDAS. Multiple cluster validation metrics based on Figures Of Merit (FOM[22,23]) have also been implemented in the software, for selection of the best-performing clustering algorithm and parameters in a given dataset. More details about GEDAS and its use are provided in a manual, which is available together with the software.

### Comparative analysis of FLAME performances: expression partitioning

To assess the performance of FLAME and compare it with the other above-mentioned algorithms, we used GEDAS to cluster four different datasets: (i) Reduced Pheripheral Blood Monocytes (RPBM) dataset [5], (ii) yeast cell cycle (YCC) expression dataset [24], (iii) hypoxia response (HR) dataset[25], and (iv) mouse tissues (MT) dataset [26]. Further details on data processing and clustering are provided in the Methods section.

**Figure 2:**
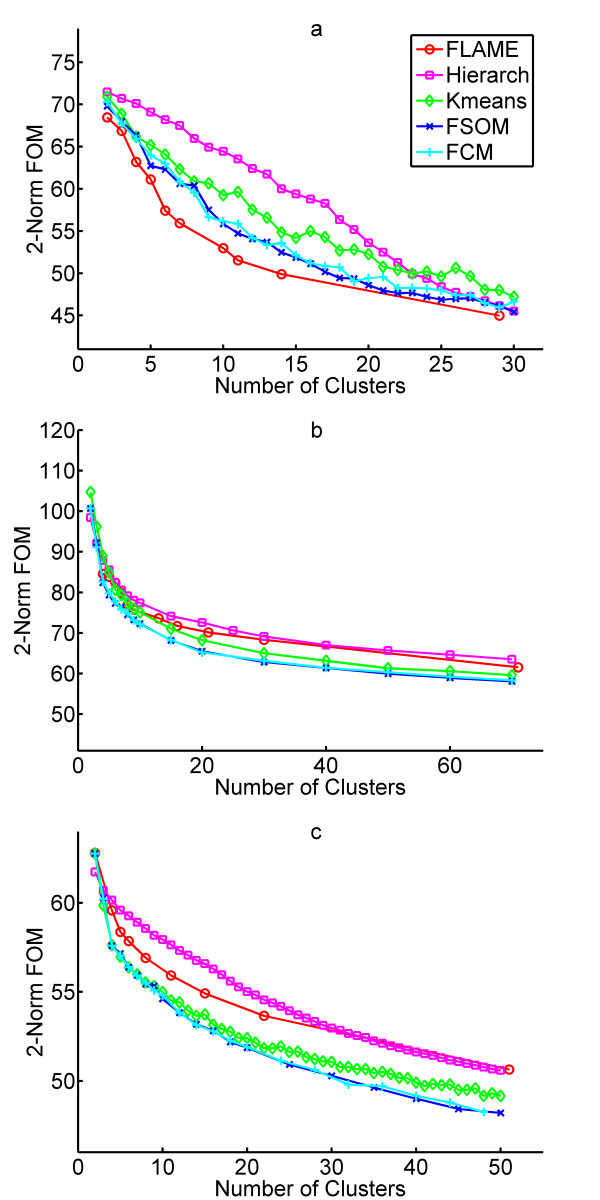
Clustering validation and comparison by 2-Norm FOM. **a**, 2-Norm FOM on the reduced peripheral blood monocyte dataset. **b**, 2-Norm FOM on the reduced hypoxia response dataset. **c**, 2-Norm FOM on the reduced yeast cell cycle dataset.

The clustering performance was initially assessed using three different Figures Of Merit (FOM)[22,23]: 1-Norm FOM, 2-Norm FOM and Range FOM (a short description of FOMs and their properties is provided in **Methods)**. We noticed that FOM analysis can not be applied to FLAME in the standard way, because there is no parameter in FLAME to directly fix the number of clusters: the cluster number is indirectly determined by the number of K-nearest neighbors (KNN) chosen. Moreover, for the same KNN number, when one experimental condition is left out during the analysis, the number of clusters generated by FLAME may change. Therefore, when applying FOM to FLAME, we use the median number of clusters generated by a given KNN during the leave-one-out analysis as the representative cluster number. The FOM analysis could not be performed on the MT dataset, because of its high sample diversity. 1-Norm FOM produced results very similar (in the sense of relative performance between algorithms) to the more widely used 2-Norm FOM (not shown). 2-Norm FOM analysis (Fig. 2) indicated that no clustering algorithm was the best in all datasets, with FLAME, hierarchical and FSOM being the best in, respectively, RPBM, HR and RYCC data. Conversely, Range FOM highlighted a better performance for hierarchical clustering in all datasets, with FLAME being the second best [see Additional file 4]. To validate clustering performance also on large datasets with reasonable computing time, we defined another validation index, named Partitioning Index, which does not require leave-one-out analysis and is defined as the ratio between the overall within-cluster variability and the overall between-cluster distance. According to this metric, a good data clustering results in low variability within each cluster and high distance between the various clusters. To calculate the overall within-cluster variability, the variability within each cluster is determined as the average distance between each pair of genes in the cluster, and then averaged for all clusters. The between-cluster distance is obtained by averaging all pairwise distances between clusters. In turn, each single between-cluster distance is calculated by averaging the distance between each pair of genes from the two clusters.

**Figure 3:**
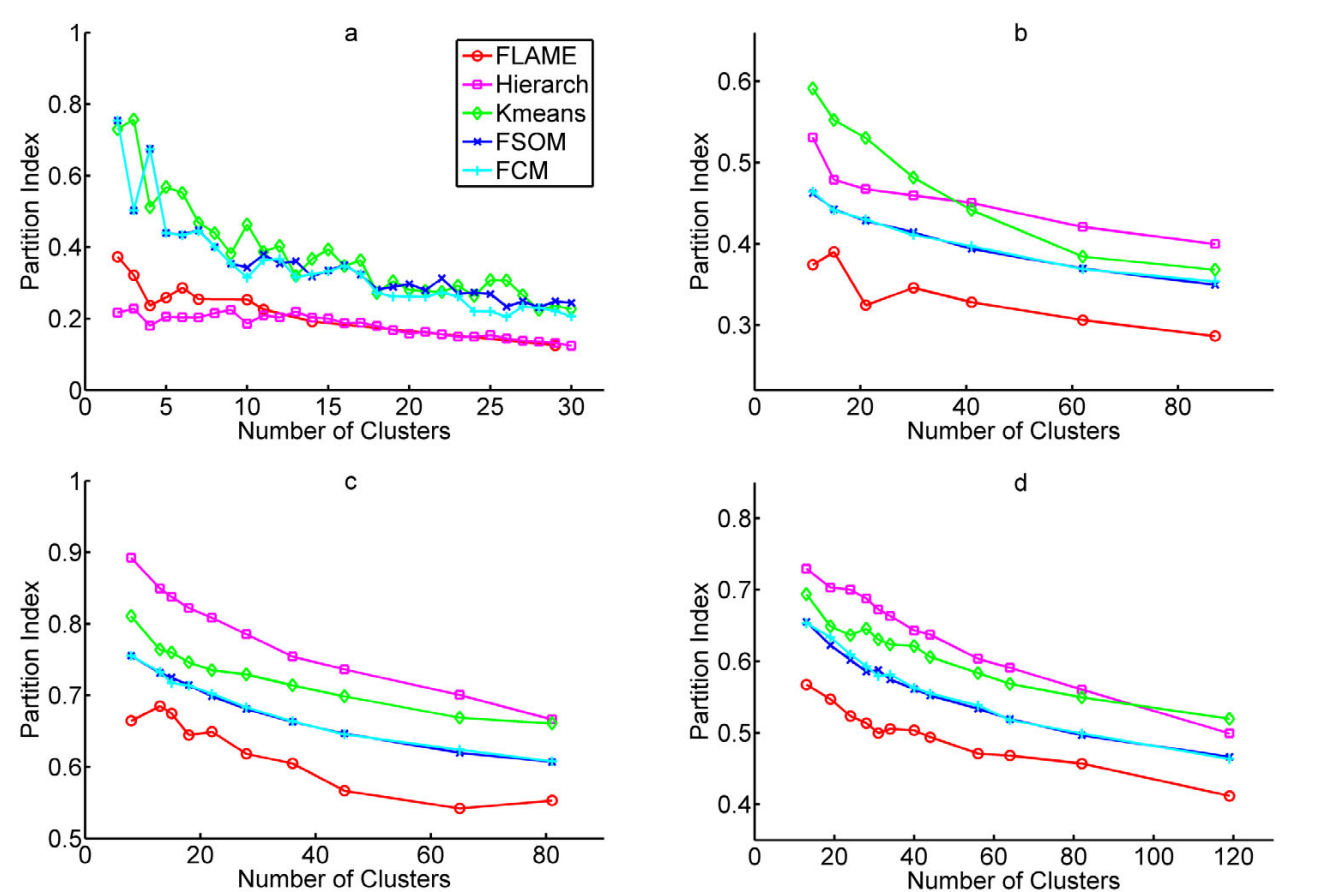
Clustering validation and comparison by Partition Index. **a**, Partition Index on the reduced peripheral blood monocyte dataset. **b**, Partition Index on the hypoxia response dataset. **c**, Partition Index on the yeast cell cycle dataset. **d**, Partition Index on the mouse tissue dataset.

Interestingly, according to the Partition Index analysis, FLAME emerged as the best algorithm in three out of four datasets (Fig. 3). A possible explanation for the different results obtained with the Partition Index analysis is that FLAME may generate non-globular clusters with more heterogeneous size distribution. Indeed, FOM is calculated by averaging the deviations in the left-out condition not cluster by cluster, but by averaging over the whole dataset. Therefore, large clusters with high internal variability have a higher weight in FOM calculation than small, compact clusters. We verified that a modified FOM calculation, where deviations are averaged at the cluster level, gives better values for FLAME (data not shown).

### Comparative analysis of FLAME performances on function partitioning

**Figure 4:**
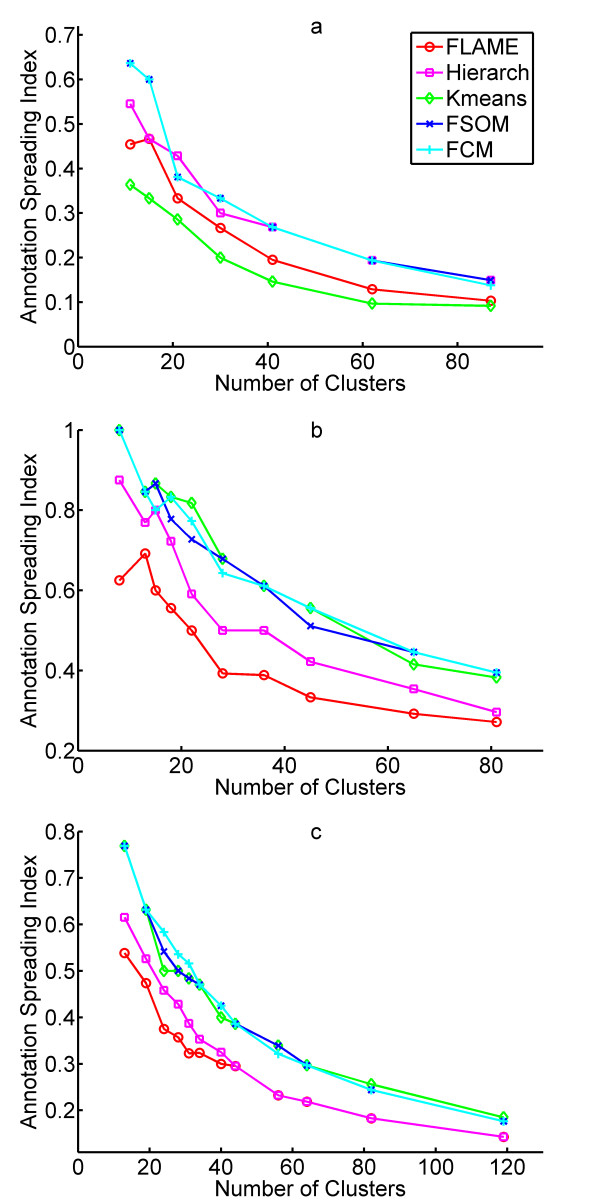
Clustering validation and comparison by Annotation Spreading Index. **a**, Spreading Index on the hypoxia response dataset. **b**, Spreading Index on the yeast cell cycle dataset. **c**, Spreading Index on the mouse tissue dataset.

As a consequence of partitioning of genes according to their expression, a good clustering algorithm should also generate clusters of functional significance, i.e. of genes that share both similar expression profiles and similar functional roles [26]. A particular caution should however be taken when using gene clustering for functional analysis, as the assumption that genes sharing the same expression profile have a similar function does not always hold true and requires extensive statistical validation [27]. To assess whether FLAME is better than other algorithms at partitioning genes into functionally homogeneous groups, we used Gene Ontology (GO) annotation [28] for a comparative assessment on three datasets (functional annotation analysis is not feasible for the RPBM dataset, as explained in Methods). For GO-based comparison, the first thing we investigated is how the GO terms are spread among the expression clusters. The rationale is that a good clustering algorithm should highlight which gene functional classes (GO terms) display a precise pattern of transcriptional regulation in a given dataset. For such classes, the algorithm should generate few expression clusters annotated with the respective GO term and many clusters without annotation to that term. A high spreading of all GO terms across the various clusters is an index of poor performance. We therefore calculated, for each GO term, the percentage of clusters with at least one annotation to that term, and defined a global Annotation Spreading Index as the median of such percentages across all GO terms. As shown in Figure 4, FLAME has a substantially lower Annotation Spreading Index in two out of three datasets.

**Figure 5:**
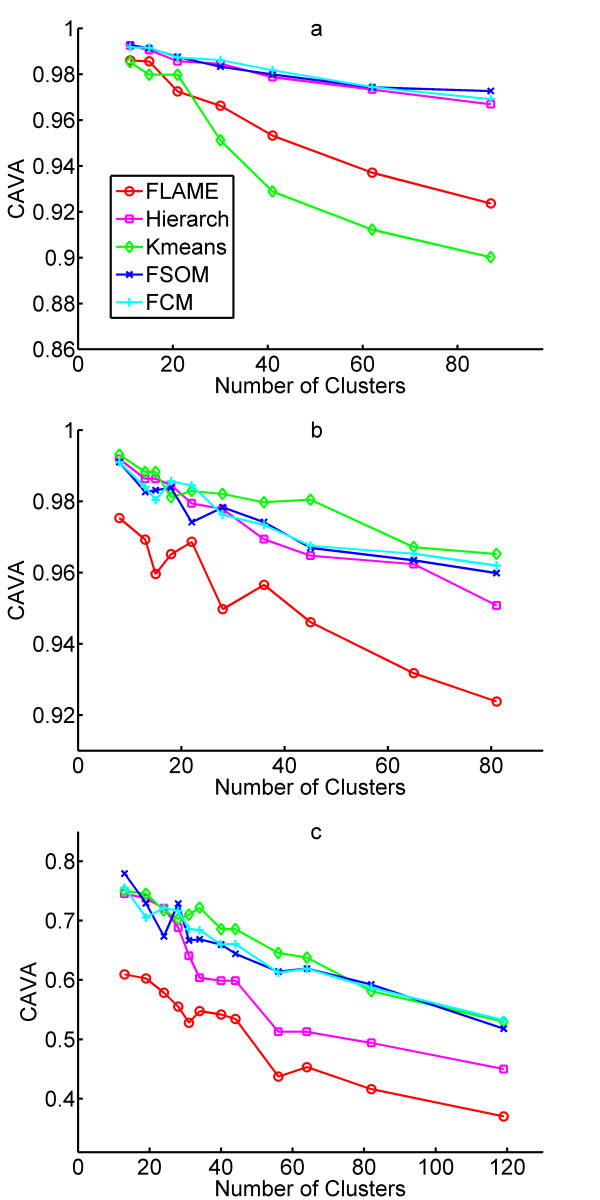
Clustering validation and comparison by Correlation to Average Annotation Profile (CAVA). **a**, CAVA on the hypoxia response dataset. **b**, CAVA on the yeast cell cycle dataset. **c**, CAVA on the mouse tissue dataset.

A second metric to assess function partitioning is based on the principle that a good clustering method should generate clusters with asymmetric distribution of functional classes, in which specific groups of functions are enriched in specific clusters. To evaluate this, we calculated a vector composed of the number of occurrences of each of the represented GO terms across the entire gene set. This vector was called the *Average Annotation Profile*. A similar vector was then calculated for each expression cluster, the *Cluster Annotation Profile *(for annotation profile matrices, see Additional file 5). We then calculated the correlation between the annotation profile of each cluster and the average annotation profile of the entire dataset. The median of the correlations between the annotation profile of each cluster and the average annotation profile finally yielded an index called *Correlation with Average Annotation *(CAVA). A high CAVA value indicates that the various functions are represented in the various clusters in a similar way, and therefore indicates poor function partitioning. As shown in Figure 5, the annotation profiles of clusters generated by FLAME display the lowest correlation to the average annotation profile in two out of three datasets. Hypergeometric distribution analysis indicated that the enrichment of GO terms in clusters generated by FLAME reached statistical significance (not shown).

In both types of analysis, we noticed that some GO terms maintain a wide distribution across all clusters and do not display particular expression patterns. This is in line with the fact that not all gene functional categories are expected to be coordinately regulated at the transcriptional level in a given set of experimental conditions.

To provide a quantitative readout of the comparative analysis between the various algorithms, we defined a way to rank the algorithms in each validation analysis based on the area below the index line plots. The algorithm giving the smallest area below the index line plot was assigned a rank of 1 (the best), and the others obtain a progressively higher value (lower rank). The results of this ranking procedure, illustrated in Table 1, show that no single clustering algorithm has always the best performance in all datasets and with all validation metrics. However, FLAME proved the best in many cases and, more importantly, its "performance profile" across the various datasets and validation metrics is profoundly different from those of the other algorithms. This indicates that FLAME can be a truly alternative clustering strategy, while, as an example, FSOM and FCM, that are tightly related clustering algorithms, display an overlapping performance profile.

**Table 1:** Ranking of each clustering algorithm across all comparative validation cases

**Dataset**	**Validation case**	**FLAME**	**Hierarchical**	**K-Means**	**F-SOM**	**FCM**
RPBM	2-Norm FOM	1	5	4	2	3
	Range FOM	2	1	5	4	3
	Partition Index	2	1	5	4	3

HR	2-Norm FOM*	5	1	4	2	3
	Range FOM*	2	1	4	5	3
	Partition Index	1	4	5	2	3
	CAVA	2	1	3	4	5
	Spreading Index	2	1	3	5	4

YCC	2-Norm FOM*	4	5	3	1	2
	Range FOM*	2	1	3	4	5
	Partition Index	1	5	4	2	3
	CAVA	1	2	5	3	4
	Spreading Index	1	2	4	3	5

MT	Partition Index	1	5	4	2	3
	CAVA	1	2	5	3	4
	Spreading Index	1	2	3	4	5

* Reduced versions of the datasets were used for these FOM analyses, due to excessive computation time required for the full-sized dataset (See Methods).

## Discussion

We present here a new algorithm for clustering microarray data, FLAME, that exploits a typical feature of "real-life" biological clusters, like sheep herds and fish shoals: the behavior of one element is dictated by the behavior of its neighbors. In other fuzzy clustering algorithms, like Fuzzy C-means or Fuzzy K-means, the fuzzy memberships of data points are directly determined by their similarity to a series of calculated cluster prototypes (or centroids). Conversely, FLAME uses pairwise similarity measures only to define the neighbors of each gene and how close each gene is to its nearest neighbors, and then approximates the fuzzy memberships of each object from its neighbors' memberships. In this approach, the cluster "prototypes", that we named Cluster Supporting Objects (CSOs, See Methods section), are defined as individual genes having a particularly high number of neighbors. The behavior of such genes would therefore be an "archetypal" behavior, shared with many other genes, and therefore likely to correctly represent the data structure. After defining the CSOs, the membership approximation propagates like a wave from the CSOs to other far objects through a network formed by the neighborhood relationships. In this way FLAME, essentially, performs the clustering using not the expression data, but the local information extracted from them, which allows reliable capturing of both linear and non-linear relationships.

In some sense, FLAME is also a kind of self-organization method. However, this self-organization process is quite distinct from the one of Self-Organizing Maps (SOM) and fuzzy SOM, which is based directly on the expression measurements. SOM also defines a neighborhood, but this neighborhood is defined only for neurons (i.e. cluster prototypes), and set in advance to constrain the cluster orientations independently from the dataset. In FLAME, instead, the neighborhood relationships are calculated for all objects, and are used to constrain the fuzzy memberships with no external inputs on the cluster number and size.

In principle, the possible applications of FLAME are not limited to gene expression datasets. In particular, the assumption that neighboring objects should have similar fuzzy memberships is well described as a mathematical cost function (Local/Neighborhood Approximation Error). Minimization of this cost function renders FLAME theoretically very valuable, because the Local Approximation Error could possibly be used in combination with other clustering constraints to get new and more powerful clustering algorithms. FLAME can be applied to any dataset including category datasets if a neighborhood can be defined for each object. In fact, a set of neighborhood relationships among the objects is the minimum requirement of FLAME, since a rough similarity between neighboring objects can be estimated as the fraction of their common neighbors.

## Conclusion

FLAME is a new algorithm for microarray data clustering that brings significant improvements in the partitioning of genes based on their expression profiles. Its good performances derive from a combination of advantageous features, some of which are distinctive, like the ability to capture dataset-specific structures by defining neighborhood relations and the subsequent neighborhood approximation of fuzzy memberships, so that non-globular and non-linear clusters can also be captured and do not get fragmented by the process. In particular, it is the novelty of neighborhood approximation that makes FLAME distinct from all other clustering approaches. Other interesting features are common to fuzzy clustering algorithms, like non univocal assignment of memberships to genes and definition of outlier genes whose expression pattern does not allow reliable assignment to any cluster.

FLAME implementation into newly developed, dedicated C++ software allows to fully benefit of the properties of the algorithm, and to apply it also to large datasets using a standard PC with very short calculation time. The fine-tuning of the algorithm, available in the advanced mode, renders FLAME very flexible and capable of handling datasets of different size and expression heterogeneity with good performances. Our results also confirm that no clustering strategy is always the best for any data type, which renders the choice between different algorithms and the availability of various validation tools implemented in the GEDAS software extremely valuable during the optimization of the clustering.

## Methods

### Extraction of Local Structure Information and CSO Identification

In this step, local structure information is extracted and cluster supporting objects are identified. To do this, similarities between each pair of objects are calculated, and the nearest neighbors are identified. The similarity measures between each object and its nearest neighbors are used to estimate the density around that object and calculate a set of weights for Local Approximation of fuzzy memberships in next step. The set of densities forms a rough estimation of the distribution of the dataset, and they are used in this step to identify CSOs and possible cluster outliers. Different distance and density metrics have been implemented our software, here we describe the default ones.

The k-nearest neighbors (KNN) for each gene are defined as the k genes with highest similarity according to a given similarity measure. The weights defining how much each neighbor will contribute to approximation of the fuzzy membership of that neighbor are calculated as *W*_*xy*_, with ∑y∈KNN(x)wxy=1
 MathType@MTEF@5@5@+=feaafiart1ev1aaatCvAUfKttLearuWrP9MDH5MBPbIqV92AaeXatLxBI9gBaebbnrfifHhDYfgasaacH8akY=wiFfYdH8Gipec8Eeeu0xXdbba9frFj0=OqFfea0dXdd9vqai=hGuQ8kuc9pgc9s8qqaq=dirpe0xb9q8qiLsFr0=vr0=vr0dc8meaabaqaciaacaGaaeqabaqabeGadaaakeaadaaeqbqaaiabdEha3naaBaaaleaacqWG4baEcqWG5bqEaeqaaaqaaiabdMha5jabgIGiolabdUealjabd6eaojabd6eaojabcIcaOiabdIha4jabcMcaPaqab0GaeyyeIuoakiabg2da9iabigdaXaaa@3EEE@, from the similarities *S*_*xy *_between that gene and its nearest neighbors. The only requirement for a definition of weights is that, the neighbors that have higher similarities must have higher weights. The simplest one we use is wxy=sxy/∑z∈KNN(x)sxz
 MathType@MTEF@5@5@+=feaafiart1ev1aaatCvAUfKttLearuWrP9MDH5MBPbIqV92AaeXatLxBI9gBaebbnrfifHhDYfgasaacH8akY=wiFfYdH8Gipec8Eeeu0xXdbba9frFj0=OqFfea0dXdd9vqai=hGuQ8kuc9pgc9s8qqaq=dirpe0xb9q8qiLsFr0=vr0=vr0dc8meaabaqaciaacaGaaeqabaqabeGadaaakeaacqWG3bWDdaWgaaWcbaGaemiEaGNaemyEaKhabeaakiabg2da9iabdohaZnaaBaaaleaacqWG4baEcqWG5bqEaeqaaOGaei4la8YaaabuaeaacqWGZbWCdaWgaaWcbaGaemiEaGNaemOEaOhabeaaaeaacqWG6bGEcqGHiiIZcqWGlbWscqWGobGtcqWGobGtcqGGOaakcqWG4baEcqGGPaqkaeqaniabggHiLdaaaa@4810@ and distance measures is transformed into similarity measure before applying this definition. For correlation measures, additional transformation is applied to highlight their relative proximities.

The density of each gene is calculated as one over the average distance to the k-nearest neighbors. Subsequently, the set of CSOs (*X*_*cso*_) is defined as the set of objects with Local Maximum Density (LMAXD), i.e., with a density higher than that of all objects in their neighborhood. The higher *k *is, the less CSO will be identified, as a consequence the less cluster will be generated.

To define possible cluster outliers, a density threshold can be applied, so that objects with a density below the threshold are defined as possible outliers (genes with "atypical" behavior). This enables starting the clustering process from the entire dataset or after just a minimal filtering. A definition similar to LMAXD can also be applied to define outliers, namely, objects with Local Minimum Density(LMIND). In our validation, we used LMIND plus a density threshold defined by the mean minus two times standard deviation of the densities.

### Local Approximation of Fuzzy Membership

In fuzzy clustering, each object *x *is associated with a membership vector *p*(*x*), in which each element *p_i_*(*x*) indicates the membership degree of *x *in cluster *i*:

*x *: *p*(*x*) = (*p*_1_(*x*), *p*_2_(*x*),..., *p*_*M*_(*x*)),

where:

0≤pi(x)≤1;∑i=1Mpi(x)=1;
 MathType@MTEF@5@5@+=feaafiart1ev1aaatCvAUfKttLearuWrP9MDH5MBPbIqV92AaeXatLxBI9gBaebbnrfifHhDYfgasaacH8akY=wiFfYdH8Gipec8Eeeu0xXdbba9frFj0=OqFfea0dXdd9vqai=hGuQ8kuc9pgc9s8qqaq=dirpe0xb9q8qiLsFr0=vr0=vr0dc8meaabaqaciaacaGaaeqabaqabeGadaaakeaacqaIWaamcqGHKjYOcqWGWbaCdaWgaaWcbaGaemyAaKgabeaakiabcIcaOiabdIha4jabcMcaPiabgsMiJkabigdaXiabcUda7maaqahabaGaemiCaa3aaSbaaSqaaiabdMgaPbqabaGccqGGOaakcqWG4baEcqGGPaqkcqGH9aqpcqaIXaqmcqGG7aWoaSqaaiabdMgaPjabg2da9iabigdaXaqaaiabd2eanbqdcqGHris5aaaa@48E6@

and *M *= |*X_cso_*| + 1.

Note that |...| means the number of elements in a set. Each element of membership vector takes value between 0 and 1, indicating how much percentage a object belonging to a cluster, or being an outlier (the last element stands for outliers).

In FLAME, such membership vector is assigned to each object through an iterative process of local approximation. More precisely speaking, the membership vector of one object is approximated by a combination of its nearest neighbors' memberships, namely, *p*(*x*) ≈ ∑y∈KNN(x)wxyp(y)
 MathType@MTEF@5@5@+=feaafiart1ev1aaatCvAUfKttLearuWrP9MDH5MBPbIqV92AaeXatLxBI9gBaebbnrfifHhDYfgasaacH8akY=wiFfYdH8Gipec8Eeeu0xXdbba9frFj0=OqFfea0dXdd9vqai=hGuQ8kuc9pgc9s8qqaq=dirpe0xb9q8qiLsFr0=vr0=vr0dc8meaabaqaciaacaGaaeqabaqabeGadaaakeaadaaeqbqaaiabdEha3naaBaaaleaacqWG4baEcqWG5bqEaeqaaOGaemiCaaNaeiikaGIaemyEaKNaeiykaKcaleaacqWG5bqEcqGHiiIZcqWGlbWscqWGobGtcqWGobGtcqGGOaakcqWG4baEcqGGPaqkaeqaniabggHiLdaaaa@4199@, where the sum is over *x*'s nearest neighbors. And *w*_*xy*_, with ∑y∈KNN(x)wxy=1
 MathType@MTEF@5@5@+=feaafiart1ev1aaatCvAUfKttLearuWrP9MDH5MBPbIqV92AaeXatLxBI9gBaebbnrfifHhDYfgasaacH8akY=wiFfYdH8Gipec8Eeeu0xXdbba9frFj0=OqFfea0dXdd9vqai=hGuQ8kuc9pgc9s8qqaq=dirpe0xb9q8qiLsFr0=vr0=vr0dc8meaabaqaciaacaGaaeqabaqabeGadaaakeaadaaeqbqaaiabdEha3naaBaaaleaacqWG4baEcqWG5bqEaeqaaaqaaiabdMha5jabgIGiolabdUealjabd6eaojabd6eaojabcIcaOiabdIha4jabcMcaPaqab0GaeyyeIuoakiabg2da9iabigdaXaaa@3EEE@, are the weights calculated from the original dataset as described before.

The iteration proceeds to minimize the overall difference between membership vectors and their approximations, described as the Local (Neighborhood) Approximation Error,

E({p})=∑x∈X‖p(x)−∑y∈KNN(x)wxyp(y)‖2,     (1)
 MathType@MTEF@5@5@+=feaafiart1ev1aaatCvAUfKttLearuWrP9MDH5MBPbIqV92AaeXatLxBI9gBaebbnrfifHhDYfgasaacH8akY=wiFfYdH8Gipec8Eeeu0xXdbba9frFj0=OqFfea0dXdd9vqai=hGuQ8kuc9pgc9s8qqaq=dirpe0xb9q8qiLsFr0=vr0=vr0dc8meaabaqaciaacaGaaeqabaqabeGadaaakeaacqWGfbqrcqGGOaakcqGG7bWEcqWGWbaCcqGG9bqFcqGGPaqkcqGH9aqpdaaeqbqaamaafmaabaGaemiCaaNaeiikaGIaemiEaGNaeiykaKIaeyOeI0YaaabuaeaacqWG3bWDdaWgaaWcbaGaemiEaGNaemyEaKhabeaakiabdchaWjabcIcaOiabdMha5jabcMcaPaWcbaGaemyEaKNaeyicI4Saem4saSKaemOta4KaemOta4KaeiikaGIaemiEaGNaeiykaKcabeqdcqGHris5aaGccaGLjWUaayPcSdWaaWbaaSqabeaacqaIYaGmaaaabaGaemiEaGNaeyicI4SaemiwaGfabeqdcqGHris5aOGaeiilaWIaaCzcaiaaxMaadaqadaqaaiabigdaXaGaayjkaiaawMcaaaaa@5E93@

where each term is the difference between the membership vector *p*(*x*), and the linear approximation of *p*(*x*) by its neighbors ∑y∈KNN(x)wxyp(y)
 MathType@MTEF@5@5@+=feaafiart1ev1aaatCvAUfKttLearuWrP9MDH5MBPbIqV92AaeXatLxBI9gBaebbnrfifHhDYfgasaacH8akY=wiFfYdH8Gipec8Eeeu0xXdbba9frFj0=OqFfea0dXdd9vqai=hGuQ8kuc9pgc9s8qqaq=dirpe0xb9q8qiLsFr0=vr0=vr0dc8meaabaqaciaacaGaaeqabaqabeGadaaakeaadaaeqbqaaiabdEha3naaBaaaleaacqWG4baEcqWG5bqEaeqaaOGaemiCaaNaeiikaGIaemyEaKNaeiykaKcaleaacqWG5bqEcqGHiiIZcqWGlbWscqWGobGtcqWGobGtcqGGOaakcqWG4baEcqGGPaqkaeqaniabggHiLdaaaa@4199@.

In FLAME, Eq(1) is minimized to calculate a set of memberships vectors under some constraints (in addition to the natural constraints on fuzzy membership vectors) derived in the first step, that is, fixing membership vectors of CSOs and outliers to avoid the trivial solutions where all *p*(*x*) are the same.

For CSOs, each of them represents a cluster, and is assigned with an unique membership vector, where only the element with index corresponding to its own cluster is 1, all others 0. For Cluster Outliers, all of them are assigned with the same membership vector, in which the last element is 1 and others 0, For all other objects(for convenience, they are referred as X˜
 MathType@MTEF@5@5@+=feaafiart1ev1aaatCvAUfKttLearuWrP9MDH5MBPbIqV92AaeXatLxBI9gBaebbnrfifHhDYfgasaacH8akY=wiFfYdH8Gipec8Eeeu0xXdbba9frFj0=OqFfea0dXdd9vqai=hGuQ8kuc9pgc9s8qqaq=dirpe0xb9q8qiLsFr0=vr0=vr0dc8meaabaqaciaacaGaaeqabaqabeGadaaakeaacuWGybawgaacaaaa@2DF4@ = *X*\*X*_*CSO*_\*X*_*Outlier*_, their membership vectors are initialized to be the same for convenience, and all elements in each vectors have the same value, i.e. 1/M. This means in the beginning, they are uncertain which clusters they belong to. In fact, random initialization doesn't change the final result, but slightly increase the computational time.

Now we can fix the memberships of CSOs *X*_*cso *_and outliers *X*_*outlier *_as a set of constraints, and minimize eq(1). To get a simpler algorithm, we excluded and from the sum in eq(1), so we have

E˜({p})=∑x∈X˜‖p(x)−∑y∈KNN(x)wxyp(y)‖2,     (2)
 MathType@MTEF@5@5@+=feaafiart1ev1aaatCvAUfKttLearuWrP9MDH5MBPbIqV92AaeXatLxBI9gBaebbnrfifHhDYfgasaacH8akY=wiFfYdH8Gipec8Eeeu0xXdbba9frFj0=OqFfea0dXdd9vqai=hGuQ8kuc9pgc9s8qqaq=dirpe0xb9q8qiLsFr0=vr0=vr0dc8meaabaqaciaacaGaaeqabaqabeGadaaakeaacuWGfbqrgaacaiabcIcaOiabcUha7Hqabiab=bhaWjabc2ha9jabcMcaPiabg2da9maaqafabaWaauWaaeaacqWFWbaCcqGGOaakcqWG4baEcqGGPaqkcqGHsisldaaeqbqaaiabdEha3naaBaaaleaacqWG4baEcqWG5bqEaeqaaOGae8hCaaNaeiikaGIaemyEaKNaeiykaKcaleaacqWG5bqEcqGHiiIZcqWGlbWscqWGobGtcqWGobGtcqGGOaakcqWG4baEcqGGPaqkaeqaniabggHiLdaakiaawMa7caGLkWoadaahaaWcbeqaaiabikdaYaaaaeaacqWG4baEcqGHiiIZcuWGybawgaacaaqab0GaeyyeIuoakiabcYcaSiaaxMaacaWLjaWaaeWaaeaacqaIYaGmaiaawIcacaGLPaaaaaa@5EB1@

It can be prove in a heuristic way [see Additional file 2] that, E˜
 MathType@MTEF@5@5@+=feaafiart1ev1aaatCvAUfKttLearuWrP9MDH5MBPbIqV92AaeXatLxBI9gBaebbnrfifHhDYfgasaacH8akY=wiFfYdH8Gipec8Eeeu0xXdbba9frFj0=OqFfea0dXdd9vqai=hGuQ8kuc9pgc9s8qqaq=dirpe0xb9q8qiLsFr0=vr0=vr0dc8meaabaqaciaacaGaaeqabaqabeGadaaakeaacuWGfbqrgaacaaaa@2DCE@({*p*}) can be minimized by the iterative procedure defined as,

pt+1(x)=∑y∈KNN(x)wxypt(y)forx∈X˜,     (3)
 MathType@MTEF@5@5@+=feaafiart1ev1aaatCvAUfKttLearuWrP9MDH5MBPbIqV92AaeXatLxBI9gBaebbnrfifHhDYfgasaacH8akY=wiFfYdH8Gipec8Eeeu0xXdbba9frFj0=OqFfea0dXdd9vqai=hGuQ8kuc9pgc9s8qqaq=dirpe0xb9q8qiLsFr0=vr0=vr0dc8meaabaqaciaacaGaaeqabaqabeGadaaakeaafaqabeqadaaabaGaemiCaa3aaWbaaSqabeaacqWG0baDcqGHRaWkcqaIXaqmaaGccqGGOaakcqWG4baEcqGGPaqkcqGH9aqpdaaeqbqaaiabdEha3naaBaaaleaacqWG4baEcqWG5bqEaeqaaOGaemiCaa3aaWbaaSqabeaacqWG0baDaaaabaGaemyEaKNaeyicI4Saem4saSKaemOta4KaemOta4KaeiikaGIaemiEaGNaeiykaKcabeqdcqGHris5aOGaeiikaGIaemyEaKNaeiykaKcabaGaeeOzayMaee4Ba8MaeeOCaihabaGaemiEaGNaeyicI4SafmiwaGLbaGaaaaGaeiilaWIaaCzcaiaaxMaadaqadaqaaiabiodaZaGaayjkaiaawMcaaaaa@5962@

starting from *p*^0^(*x*) satisfying ∑i=1Mpi0(x)=1
 MathType@MTEF@5@5@+=feaafiart1ev1aaatCvAUfKttLearuWrP9MDH5MBPbIqV92AaeXatLxBI9gBaebbnrfifHhDYfgasaacH8akY=wiFfYdH8Gipec8Eeeu0xXdbba9frFj0=OqFfea0dXdd9vqai=hGuQ8kuc9pgc9s8qqaq=dirpe0xb9q8qiLsFr0=vr0=vr0dc8meaabaqaciaacaGaaeqabaqabeGadaaakeaadaaeWbqaaiabdchaWnaaDaaaleaacqWGPbqAaeaacqaIWaamaaaabaGaemyAaKMaeyypa0JaeGymaedabaGaemyta0eaniabggHiLdGccqGGOaakcqWG4baEcqGGPaqkcqGH9aqpcqaIXaqmaaa@3C61@. In this way, the fuzzy membership of one object in approximation cycle *t+1 *is updated by a linear combination of the fuzzy memberships of its neighbors in cycle *t*. As in the step identifying CSO and Outliers, a new neighborhood can be defined, or simply use one of the neighborhoods defined in previous steps. The combination weight *w*_*xy *_is define by the relative proximity of *y *to *x *with respect to the other neighbors of *x*, the closer *y *is to **x**, the bigger is *w*_*xy*_. The types of neighborhood and *w*_*xy *_effect the fuzziness of the clustering. For t → ∞, *p*^*t*^(*x*) will converge to p* with E˜
 MathType@MTEF@5@5@+=feaafiart1ev1aaatCvAUfKttLearuWrP9MDH5MBPbIqV92AaeXatLxBI9gBaebbnrfifHhDYfgasaacH8akY=wiFfYdH8Gipec8Eeeu0xXdbba9frFj0=OqFfea0dXdd9vqai=hGuQ8kuc9pgc9s8qqaq=dirpe0xb9q8qiLsFr0=vr0=vr0dc8meaabaqaciaacaGaaeqabaqabeGadaaakeaacuWGfbqrgaacaaaa@2DCE@({p*}) = 0. And in each step, *p*^*t*^(*x*) satisfy ∑i=1Mpit(x)=1
 MathType@MTEF@5@5@+=feaafiart1ev1aaatCvAUfKttLearuWrP9MDH5MBPbIqV92AaeXatLxBI9gBaebbnrfifHhDYfgasaacH8akY=wiFfYdH8Gipec8Eeeu0xXdbba9frFj0=OqFfea0dXdd9vqai=hGuQ8kuc9pgc9s8qqaq=dirpe0xb9q8qiLsFr0=vr0=vr0dc8meaabaqaciaacaGaaeqabaqabeGadaaakeaadaaeWbqaaiabdchaWnaaDaaaleaacqWGPbqAaeaacqWG0baDaaaabaGaemyAaKMaeyypa0JaeGymaedabaGaemyta0eaniabggHiLdGccqGGOaakcqWG4baEcqGGPaqkcqGH9aqpcqaIXaqmaaa@3CE4@. The set of outliers can be enlarged after the Neighborhood Approximation of Fuzzy Membership, due to the fact that some other objects will have similar memberships as outliers.

### Cluster Construction

When a set of fuzzy memberships is calculated, clusters can be defined based on a one-to-one gene-cluster assignment. Alternatively, one object can be assigned to more than one cluster if it has a reasonably high membership score for multiple clusters. Also, some objects may not be assigned to any clusters if they don't have one dominant membership percentage. The objects not assigned to any cluster are regarded as outliers. In this way more objects can be screened out from clusters.

### Figures of Merit

The use of Figures of Merit (FOMs) has been proposed by Yeung and colleagues [22,23] to characterize the predictive power of different clustering algorithms. FOM is estimated by removing one experiment at a time from the dataset, clustering genes based on the remaining data, and then measuring the within-cluster similarity of the expression values in the left-out experiment. The principle is that correctly co-clustered genes should retain a similar expression level also in the left-out sample. The assumption (and limit) of this approach is that most samples have correlated gene expression profiles. The most commonly used FOM, referred to as "2-Norm FOM" [22], measures the within-cluster similarity as root mean square deviation from the cluster mean in the left-out condition. Then, an aggregated FOM is obtained by summing up all the FOMs of all left-out experiments and used to compare the performance of different clustering algorithms (the lower the FOM, the better the predictive power of a clustering algorithm). Other types of FOM measure the within-cluster similarity in different ways [23]. Of these, "1-Norm FOM" and "Range FOM" have also been used in this work. 1-Norm FOM measures the within-cluster similarity in the left-out experiment as the average of the Manhattan distances between the expression levels of genes and the mean expression level in the clusters. Range FOM is the average difference between the maximum and minimum expression levels in the clusters in the left-out experiment. While 1-Norm and 2-Norm FOM measure the compactness of clusters, Range FOM measures the diameter of clusters regardless the distribution of expression values in a cluster. Different FOM may favor different clustering algorithms dependent on the clustering criteria employed by them. Moreover, FOM may not be applied to datasets where most of the experimental conditions display highly divergent gene expression profiles [4].

### Datasets and analysis parameters

#### RPBM

It is a reduced version of a Pheripheral Blood Monocytes dataset originally used by Hartuv et al. to test their clustering algorithm [29]. The dataset consists of 139 hybridizations (performed with 139 different oligonucleotide probes) on an array of 2329 spotted cDNAs derived from 18 genes. The rationale is that spotted cDNAs derived from the same gene should display a similar profile of hybridization to the 139 probes and therefore be clustered together. This dataset was then reduced to contain 235 cDNAs only by Gesu et al. [5] to reduce the computational time for applying FOM analysis. No validation based on gene functional annotation can be performed on this dataset since it does not reflect gene expression.

#### YCC & RYCC

They yeast cell cycle data is a part of the studies by Spellman et al. [24]. The complete dataset contains about 6178 genes and 76 experimental conditions. The reduced yeast cell cycle (RYCC) dataset is a subset of the original YCC dataset being selected by Yeung et al for FOM analysis [22,23] and composed of 698 genes and 72 experimental conditions. To facilitate functional annotation analysis, we processed from the complete data and got a subset of 5529 genes which are annotated to a total number of 64 biological process GO terms at level 4.

#### HR & RHR

The hypoxia response (HR) dataset is a part of the recent work conducted by Chi et al. [25] to investigate cell type specificity and prognostic significance of gene expression programs in response to hypoxia in human cancers. The dataset is downloaded from Stanford Microarray Database[30] with default filtering parameters provided by the web interface. This initial dataset includes 11708 genes and 57 experimental conditions. Then the genes in the dataset is annotated to GO terms and only genes with annotation to biological process GO terms are selected for further analysis. This results in a subset of 6613 genes. Noticing that some of the genes has null values in most of the experimental conditions, genes with more than 80% null values are filtered out to facilitate the clustering analysis. This final dataset includes 6029 genes. In the end the GO terms are also mapped to level 4, resulting 149 GO terms. A subset (RHR, reduce hypoxia response dataset) of the final dataset was created for FOM analysis by selection the top 1000 genes with the highest expression variations.

#### MT

The mouse tissue (MT) data is the result generated from the work of Zhang et al. [26] on 55 mouse tissues, including 21622 confidently detected transcripts. We analyzed a subset of 6831 transcripts annotated with the 230 Gene Ontology (GO) terms defined by Zhang and colleagues [26] as "super GO" terms. FOM-based analysis was not performed on this dataset due to the fact that it contains individual tissue samples from many different organs with highly diverse expression profiles, which make the basic assumption of FOM not valid in this dataset.

#### Parameters

Cosine correlation was used as a distance metric for all datasets except MT, for which centered Pearson was used. All other parameters except the one to determine the cluster number for each method used the default values implemented in the GEDAS software: un-weighted pair-group average linkage for hierarchical clustering; 500 as maximum iteration number and 1E-6 as the converging criteria for all methods except hierarchical clustering. Clusters with a large range of cluster numbers were generated for the comparison analysis. Depending on the datasets and the type of analysis, different ranges of K-nearest numbers for FLAME had to be used to generate a reasonable range of cluster numbers. Then the parameter to determine the cluster number for other methods were chosen to generate the same or similar number of clusters.

## Availability and requirements

**Project Name: **Gene Expression Data Analysis Studio

**Project Home Page: **

**Operating Systems: **OS Portable (Source code to work with many OS platforms)

**Programming Language: **C++

**Other Requirements: **Need Qt4 Library (available from Trolltech [31])

**License: **GNU General Public License (GPL)

## Authors' contributions

LF designed the FLAME algorithm and implemented it in the GEDAS software, and wrote scripts to perform the post-clustering analysis. EM contributed to the refining of parts of the algorithm and the post-clustering analysis. All the authors have read and approved the final manuscript.

## Supplementary Material

Additional file 1 Animation to Demonstrate Membership Propagation. This animation shows how the influence of memberships of CSOs and outliers propagate through a network formed by neighborhood relationships, and how a gene gets a fuzzy membership by the balanced influence from its neighbors. Click here for file

Additional file 2 Additional Note. This note includes a proof for the heuristic optimization procedure used in FLAME and a rough time complexity analysis of the FLAME algorithm. Click here for file

Additional file 3 Empirical time complexity comparison of FLAME with other algorithms. This comparison is done on the hypoxia dataset with 57 samples. Gene subsets of different sizes are obtained by choosing genes with the highest variations. Click here for file

Additional file 4 Clustering validation and comparison by range FOM. **a**, range FOM on the reduced peripheral blood monocyte dataset. **b**, range FOM on the reduced hypoxia response dataset. **c**, range FOM on the reduced yeast cell cycle dataset. Click here for file

Additional file 5 Annotation matrices. Annotation matrices of 44 clusters (rows) across 230 GO terms (columns) obtained from the mouse tissue dataset. The color scale indicates the number of counts for each GO term (column) in each cluster (row). Matrices obtained by FLAME, hierarchical, k-means, fuzzy SOM and fuzzy C-means clustering mouse tissue dataset are shown, as indicated. The grey color indicates zero counts for a given GO term in a given cluster. The average annotation profile can be detected as a row without grey cells and with name "dataset" in the right part. Click here for file
