# Supplementary material for: FLAME, a novel fuzzy clustering method for the analysis of DNA microarray data
Source: BMC Bioinformatics. 2007 Jan 4;8:3. doi: 10.1186/1471-2105-8-3 (PMC1774579; doi:10.1186/1471-2105-8-3)
Supplement: Additional file 2 — Additional Note. This note includes a proof for the heuristic optimization procedure used in FLAME and a rough time complexity analysis of the FLAME algorithm. [file 1471-2105-8-3-S2.pdf]

Fu and Medico, *FLAME, A novel fuzzy clustering method for the analysis of DNA microarray data*

Supplementary Note

Limin Fu

1 Proof for The Heuristic Optimization Procedure

Here we derived the heuristic iterative procedure to find the global minimum of Local (Neighborhood) Approximation Error $\widetilde{E}(\{p\})$. The optimal $\{p\}$ that minimize the Local Approximation Error $\widetilde{E}(\{p\})$ satisfy, for $\forall \mathbf{x} \in \widetilde{\mathbf{X}}$

$$\begin{aligned} 0 &= \frac{\partial\Big(\frac{1}{2}\widetilde{E}(\{p\}) - \lambda(\mathbf{x})(\sum_{k=1}^{M} p_k(\mathbf{x}) - 1)\Big)}{\partial p_k(\mathbf{x})} \\ &= p_k(\mathbf{x}) - \sum_{\mathbf{y}\in\mathbf{G_x}} w_{\mathbf{xy}} p_k(\mathbf{y}) \\ &\quad - \sum_{\{\mathbf{z}:\mathbf{x}\in\mathbf{G_z}\}} w_{\mathbf{zx}}\Big(p_k(\mathbf{z}) - \sum_{\mathbf{u}\in\mathbf{G_z}} w_{\mathbf{zu}} p_k(\mathbf{u})\Big) - \lambda(\mathbf{x}) \end{aligned} \quad (1)$$

where $\lambda(\mathbf{x})$ is the Lagrange multiplier for the constraint $\sum_{k=1}^{M} p_k(\mathbf{x}) = 1$ (M cluster number), and $\sum_{\{\mathbf{z}:\mathbf{x}\in\mathbf{G_z}\}}$ is sum over the data points which have $\mathbf{x}$ as one of their nearest neighbors. So far we didn't consider the constraints of $0 \le p_k(\mathbf{x}) \le 1$, but finally we will see that these constraints can be automatically satisfied in the heuristic optimization procedure. Since $\sum_k p_k(\mathbf{x}) = 1$, summing Eq.1 over k, we have $\lambda(\mathbf{x}) = 0$.

Now, if we denote $\delta_k(\mathbf{x}) = p_k(\mathbf{x}) - \sum_{\mathbf{y}\in\mathbf{G_x}} w_{\mathbf{xy}} p_k(\mathbf{y})$, we have,

$$\delta_k(\mathbf{x}) - \sum_{\{\mathbf{z}:\mathbf{x}\in\mathbf{G_z}\}} w_{\mathbf{zx}}\delta_k(\mathbf{z}) = 0, \; k = 1, ..., M-1 \quad (2)$$

$$\sum_{k=1}^{M} \delta_k(\mathbf{x}) = 0 \quad (3)$$

$$\forall \mathbf{x} \in \widetilde{\mathbf{X}}$$

Now note that, the coefficient matrix of the above linear equations have non-zero determinant, because this matrix can be rearranged so that the diagonal elements are 1, and no two rows and columns are correlated. So $\delta(\mathbf{x}) = \mathbf{0}$.

So now we have,

$$p_k(\mathbf{x}) - \sum_{\mathbf{y}\in\mathbf{G_x}} w_{\mathbf{xy}} p_k(\mathbf{y}) = 0, \; k = 1, ..., M-1 \quad (4)$$

$$\sum_{k=1}^{M} p_k(\mathbf{x}) = 1 \quad (5)$$

by similar argumentation, the above equations will have a unique solution. These linear equations can be solved by an iterative procedure defined by Eq.3 in the **Methods** section of the paper, or by standard techniques for solving linear equations.

2 Time Complexity of FLAME

Suppose the number of genes under clustering is N, the number of experimental conditions is M, the number of CSO (the same as the number of clusters) is C, and all the steps use the same number K of K-Nearest Neighbors. In the first step of FLAME to define nearest neighbors, for $M << N$ and $K << N$, the time complexity is

$$O(M \cdot N \cdot (N + K\log(N))) \sim O(N^2)$$

which is the time many other clustering algorithms have also to spend in the initial stage of clustering. The time spend in the second step is ignorable compared with the other two steps. In the third step for membership approximation, there is a linear equation to solve, which in general has $O(N \cdot C)^3$ computational complexity in theory. But the coefficient matrix of that equation is actually extremely sparse for large N, it is solved efficiently in our implementation by an iterative procedure. The theoretic time complexity analysis of this iterative procedure is very difficult, so an empirical study of the time complexity for FLAME is provided in additional file: Figure 1.
