# Supplementary figures and images for: FLAME, a novel fuzzy clustering method for the analysis of DNA microarray data
Source: BMC Bioinformatics. 2007 Jan 4;8:3. doi: 10.1186/1471-2105-8-3 (PMC1774579; doi:10.1186/1471-2105-8-3)

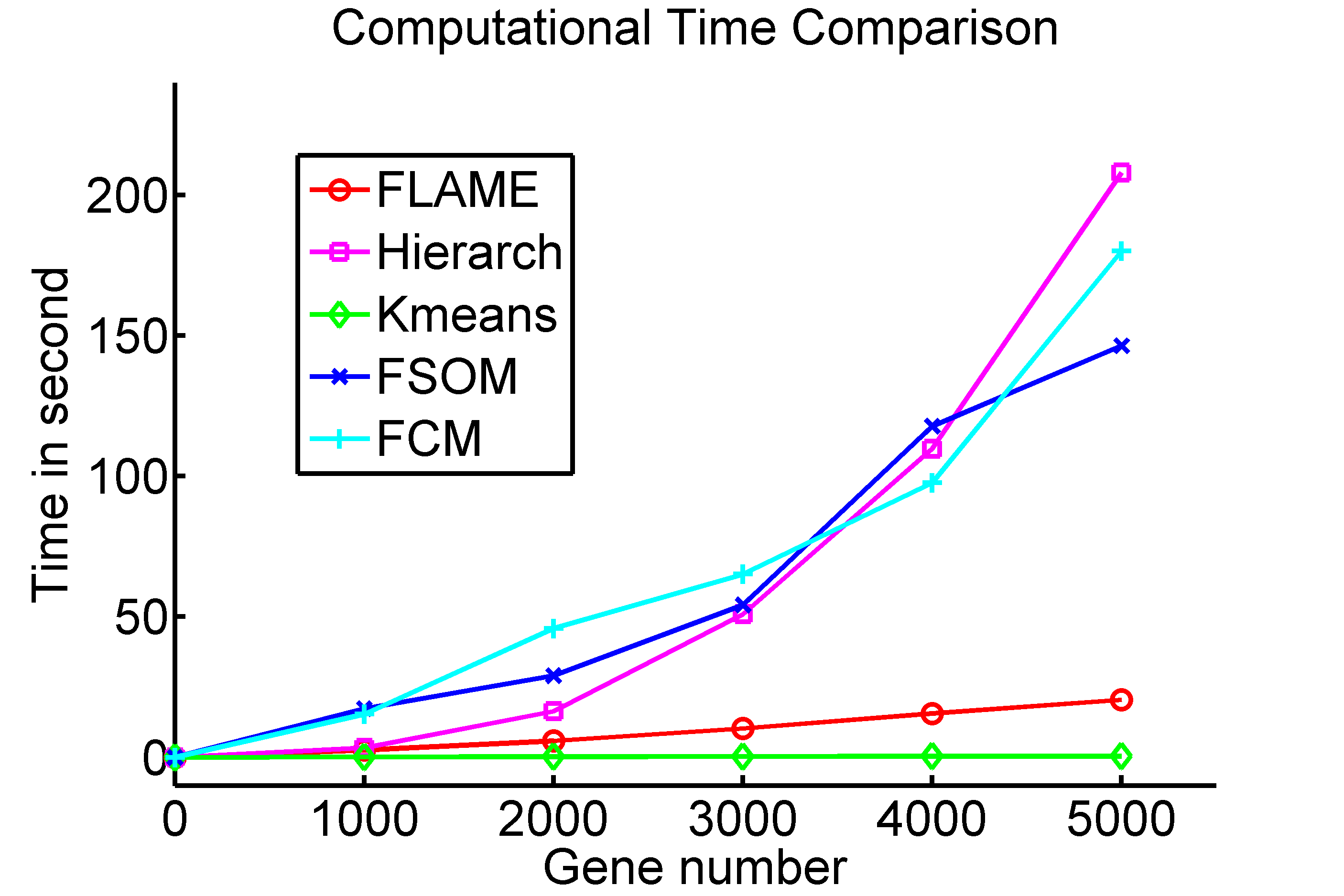

Supplement: Additional file 3 — Empirical time complexity comparison of FLAME with other algorithms. This comparison is done on the hypoxia dataset with 57 samples. Gene subsets of different sizes are obtained by choosing genes with the highest variations. [file 1471-2105-8-3-S3.png]

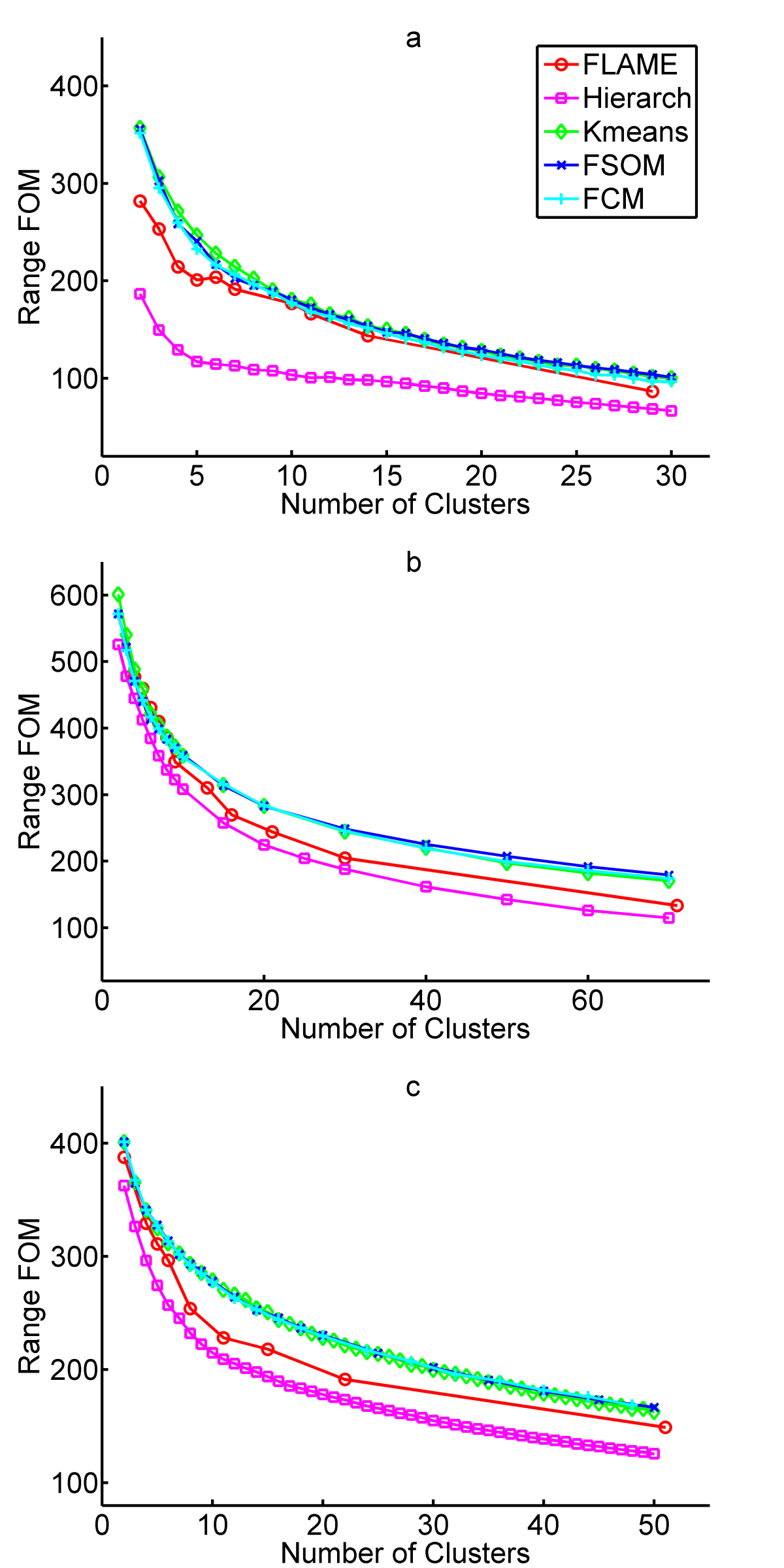

Supplement: Additional file 4 — Clustering validation and comparison by range FOM. a, range FOM on the reduced peripheral blood monocyte dataset. b, range FOM on the reduced hypoxia response dataset. c, range FOM on the reduced yeast cell cycle dataset. [file 1471-2105-8-3-S4.png]

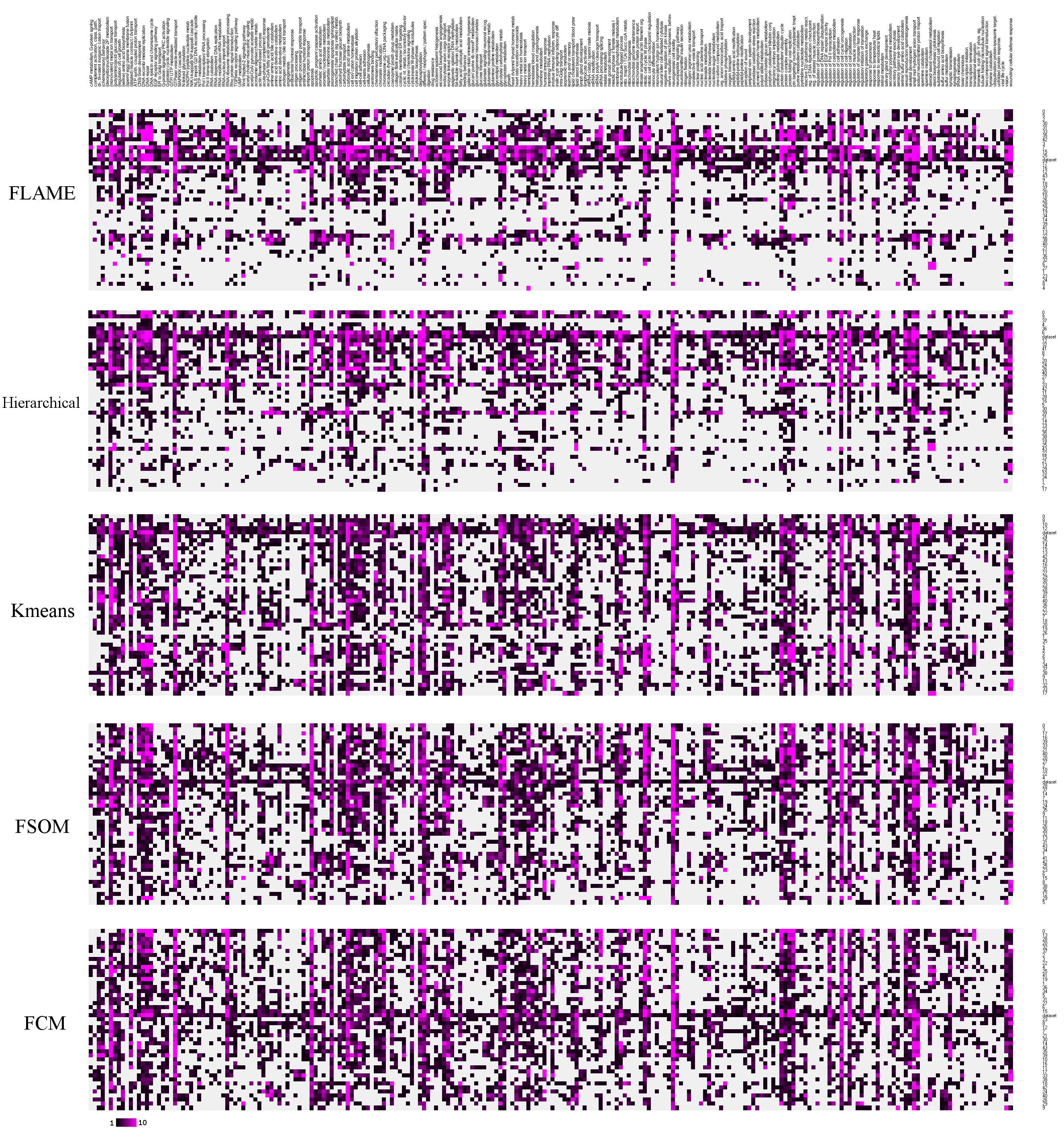

Supplement: Additional file 5 — Annotation matrices. Annotation matrices of 44 clusters (rows) across 230 GO terms (columns) obtained from the mouse tissue dataset. The color scale indicates the number of counts for each GO term (column) in each cluster (row). Matrices obtained by FLAME, hierarchical, k-means, fuzzy SOM and fuzzy C-means clustering mouse tissue dataset are shown, as indicated. The grey color indicates zero counts for a given GO term in a given cluster. The average annotation profile can be detected as a row without grey cells and with name "dataset" in the right part. [file 1471-2105-8-3-S5.png]
